# Supplementary material for: The Impact of Acetyl-CoA and Aspartate Shortages on the N-Acetylaspartate Level in Different Models of Cholinergic Neurons
Source: Antioxidants (Basel). 2020 Jun 13;9(6):522. doi: 10.3390/antiox9060522 (PMC7346116; doi:10.3390/antiox9060522)
Supplement: Supplementary file 1 [file antioxidants-09-00522-s001.pdf]

Supplement 1. Protocols for enzymatic assays

Aconitase (Aco, EC 4.2.1.3) activity was determined using NADPH / NADP conversion technique, at λ = 340 nm and 37 ^{o}C. The reaction buffer contained 0.05 M Tris-HCl (pH = 7.4), 2 mM $MgCl_2$, 0.1 mM NADP, 1 U IDH-NADP and 100 μg of cell homogenate protein in a final volume of 0.7 mL. Enzymatic assay was initiated by the addition of 10 μL of 10 mM *cis*-aconitane (10 μL) [16].
Aspartate aminotransferase (GOT, EC 2.6.1.1) activity was determined using DTNB/TNB reduction technique, at 412 nm and 37^{0}C. 1 mL of reaction buffer contained 0.1 M Tris-HCl (pH=8.3), 1 mM sodium-EDTA, 0.018 mM acetyl-CoA, 50 mM aspartate, 0.1 mM DTNB, 2U citrate synthase (SC, EC 4.1.3.7) and 20 μg of cell homogenate protein. Enzymatic assay was initiated by the addition of 10 μL of 0.1 mM α-ketoglutarate [17].
Aspartate *N*-acetyltransferase (Asp-NAT, 2.3.1.17) activity was determined using radiochemical assay to measure the level of produced [^{14}C]-*N*-acetylaspartate (^{14}C-NAA). The reaction buffer contained 10 mM potassium phosphate, 20 mM potassium-HEPES (pH=7.1), 1 mM $MgCl_2$, 200 μM acetyl-CoA, 50 μM aspartate, 1 μM L-[U-^{14}C]aspartate and 100 μg of cell homogenate protein in a final volume of 0.2 mL (incubation conditions: 30 min, 37 ^{0}C, gentle shaking). The reaction was stopped by thermic shock (5 min, 80 ^{0}C) and dilution (1 mL of 5 mM potassium-HEPES, pH=7.1). The final product (^{14}C-NAA) was isolated on DSC-SAX-SPE column (Discovery, Cat# 52664-U). Mobile phases: wash 1 (2 mL of 5 mM potassium-HEPES, (pH=7.1)), wash 2 (5 mL of 0.15 M NaCl), and elution (5 mL of 0.3 M NaCl). The radioactivity of the eluent (2 mL) was counted for 10 min in the presence of 10 mL Ultima Gold™ liquid scintillator (Perkin Elmer, Cat# 6013321). Total radioactivity of substrate (L-[U-14C]aspartate) has been measured in 1,4-dioxan-based scintillator instead of Ultima Gold™ scintillator [18].
Citrate synthase (SC, EC 4.1.3.7) activity was determined using DTNB/TNB reduction technique, at 412 nm and 37 ^{0}C. 1 mL of reaction buffer contained 0.1 M Tris-HCl (pH=8.0), 0.015 mM acetyl-CoA, 0.2 mM DTNB and 20 μg of cell homogenate protein. Enzymatic assay was initiated by the addition of 10 μL of 0.2 mM oxaloacetate [20].
Choline acetylaspartate (ChAT, EC 2.3.1.6) activity was determined using radiochemical assay to measure the level of produced [^{14}C]-acetylcholine (^{14}C-ACh). The reaction buffer contained 50 mM sodium phosphate buffer (pH = 7.4), 1 mM EDTA, 0.6 mM NaCl, 0.2 mM eserine, [1-^{14}C]-acetyl-CoA (0.43 nmol / sample, 0.025 μCi), 0.05 M choline and 20 μg of cell homogenate protein in a final volume of 0.01 mL (incubation conditions: 30 min, 30 ^{o}C, gentle shaking). Reaction was stopped by dilution in ice – cold buffer having 10 mM sodium phosphate buffer (pH = 7.4), 0.5% 4-tetraphenylborate sodium, 25% acetonitrile in total volume of 6 mL. Finally, radioactive ^{14}-C ACh has been extracted in POPOP / PPO / toluene liquid scintillator and measured for 10 min. Total radioactivity of substrate ([1-^{14}C]-acetylo-CoA) has been measured in 1,4-dioxan-based scintillator instead [19].
Glutamate dehydrogenase (GDH, EC 1.4.1.2) activity was determined using NADPH/NADP conversion technique, at 340 nm and 37 ^{0}C. 1 mL of reaction buffer contained 0.1 M Tris-HCl (pH=8.3), 3.3. M NH_4Cl, 7.5 mM NADPH and 20 μg of cell homogenate protein. Enzymatic assay was initiated by the addition of 10 μL of 0.1 M α-ketoglutarate [21].
Hexokinase (Hex, EC 2.7.1.1) activity was determined using NADPH / NADP conversion technique, at λ = 340 nm and 37 ^{o}C. 1 mL of reaction buffer contained 0.1 M Tris-HCl (pH = 7.4), 5 mM glucose, 0.2 mM $MgCl_2$, 0.25 mM NADH, 1 U glucose-6-phosphate dehydrogenase (G-6-PDH, EC 1.1.1.49) and 20 μg of cell homogenate protein. Enzymatic assay was initiated by the addition of 10 μL of 0.1 M pyruvate [22].
Isocitrate dehydrogenase (IDH, EC 1.1.1.42) activity was determined using NADPH / NADP conversion technique, at λ = 340 nm and 37 ^{o}C. The reaction buffer contained 0.05 M Tris-HCl (pH = 7.4), 0.6 mM $MgCl_2$, 0.5 mM NADP and 100 μg of cell homogenate protein in a final volume of 0.7 mL. Enzymatic assay was initiated by the addition of 10 μL of 10 mM isocitrate [23].
Lactate dehydrogenase or LDH in media assay (LDH, EC 1.1.1.27) activity was determined using NADH / NAD conversion technique, at λ = 340 nm and 37 ^{o}C. 1 mL of reaction buffer contained 0.1 M Tris-HCl (pH = 7.4), 0.2 mM NADH and 20 μg of cell homogenate protein (or 0 – 200 μL of culture

media, for cell viability test assay). Enzymatic assay was initiated by the addition of 10 μL of 0.1 M ATP. To establish total LDH in media activity, Triton X-100 in final concentration 0.2% was added to two culture dishes with untreated cells. Dishes were incubated for the additional 2 h in usual culture conditions. Eventually, LDH in media activity from each experimental time point was divided by total LDH activity and express as a per cents of total LDH activity [24].

Pyruvate dehydrogenase complex (PDHC, EC 1.2.4.1.) activity was determined using cycling method. In each lysate (100 μg of cell homogenate protein) the following reactions were performed: (1) citrate production, (2) citrate level measurement. The first reaction (1) was carried out in 250 μL for 30 min (37 ^{o}C, gentle shaking), buffer contained 0.1 M Tris-HCl (pH = 8.3), 2 mM $MgCl_2$, 10 mM dithiothreitol, 10 mM pyruvate, 2 mM thiamine pyrophosphate, 0.2 mM CoA, 2.5 mM oxaloacetate, 2 mM NAD, 0.15 U citrate synthase (EC 4.1.3.7). Reaction was terminated by thermic shock (10 min, 100 ^{o}C). Finally, the produced citrate level was determined using NADH / NAD conversion technique, at λ = 340 nm and 37 ^{o}C. The reaction buffer contained 0.1 M Tris-HCl (pH = 7.4), 0.1 mM NADH, 0.2 U malate dehydrogenase (EC 1.1.1.37) and 100 μL of achieved supernatant in a final volume of 0.7 mL. The assay was initiated by the addition of 10 μL of 0.1 U citrate lyase (EC 4.1.3.6) [25].

Malate dehydrogenase (MDH, EC 1.1.1.37) activity was determined using NADH/NAD conversion technique, at 340 nm and 37 ^{0}C. 1 mL of reaction buffer contained 0.1 M Tris-HCl (pH=7.4), 3 mM sodium-EDTA, 0.25 mM NADH and 20 μg of cell homogenate protein. Enzymatic assay was initiated by the addition of 10 μL of 0.15 M oxaloacetate [26].

Supplement 2. Protocols for metabolic assays

Acetoacetate level was determined using NADH/NAD conversion technique, at 340 nm and 37^{0}C. 1 mL of reaction buffer contained 0.1 M TRIS-HCl buffer (pH=7.4), 0.2 mM NADH and 20 µL of urine. Reaction was initiated by the 10 µL addition of 0.25 U β-hydroxybutyrate dehydrogenase [27].

Acetyl-CoA level was determined using the cycling method described previously [15]. In each neutralized supernatant (40 µg of cell homogenate protein), the following reactions were performed: (1) coenzyme-A removal, (2) acetyl-CoA level enhancement, (3) citrate level measurement. The first reaction (1) was carried out in 50 µL for 2 h (room temperature, gentle shaking). The first reaction buffer contained 0.1 M Tris-HCl (pH=7.4), 1 mM maleic anhydride (dissolved in diethyl ether). The second reaction was started by the addition of 50 µL of the second reaction buffer (50 mM Tris-HCl (pH=7.4), 5 mM NH_4Cl, 0.01% albumin, 1.2 mM oxaloacetate, 2 mM acetyl phosphate, 1 U phosphotransacetylase, and 0.12 U citrate synthase). The reaction (30 ^{0}C, gentle shaking) lasting for 100 min was terminated by thermic shock (10 min, 100 ^{0}C). Finally, the produced citrate was determined using NADH/NAD conversion technique, at 340 nm and 37 ^{0}C. The reaction buffer contained 0.1 M Tris-HCl (pH=7.4), 0.1 mM NADH, 0.2 U MDH and obtained supernatant in a final volume of 0.7 mL. The assay was initiated by the addition of 10 µL of 0.1 U citrate lyase (EC 4.1.3.6) [15].

Acetylcholine release were assayed by luminescence. After experimental step, each sample (200 µL) was spun down (5 min, 10 000 xg). Acetylcholine release was measured in a supernatant. The reaction buffer contained: 67 mM glycine-NaOH buffer (pH=8.3), 0.01 mM luminol, 10 U horseradish peroxidase (EC 1.11.1.7), 0.5 U choline oxidase (EC 1.1.3.17) and 20 µL of sample. Reaction was initiated by the 10 µL addition of 5 U acetylcholinesterase (EC 3.1.1.7) [27].

Aspartate level was determined using NADH/NAD conversion technique at 340 nm and 37 ^{0}C. The reaction buffer contained 70 mM sodium/potassium-phosphate buffer (pH=7.2), 10 mM α-ketoglutarate, 0.2 mM NADH and 100 µg of cell homogenate protein in a final volume of 0.7 mL. The assay was initiated by the addition of 10 µL of 15 U MDH and 0.6 U GOT [28].

ATP levels were assayed by RP-HPLC method. Briefly, supernatant (pH = 7.0) was centrifuged at Micro Spin filter (Teflon membrane, Ø 0.22 mm, CIRO, Cat# CIPT-02). 25 µL of supernatant (50 µg of cell homogenate protein) was analyzed in a pre-column protected Hypersil™ ODS C18RP column (150 x 4.6, i.d., MZ-Analysentechnik GmbH, Cat# 6045) by Flexar HPLC system (Perkin Elmer). Mobile phase A (10mM TBAHS / 100mM phosphate buffer, pH = 7.0) and mobile phase B (30% methanol) were mixed with flow rate: 1 mL/min under gradient program: 0 – 10 min (98% mobile phase A), 10 – 25 min (linear gradient form 98% to 0% mobile phase A), 25 – 40 min (0% mobile phase A), 40 – 45 (linear gradient form 0% to 98% mobile phase A). Separation time: t_{ATP} = 22.0 min [29].

β-hydroxybutyrate level was determined using NADH / NAD conversion technique, at λ = 340 nm and 37 ^{o}C. 1 mL of reaction buffer contained 0.08 M glycine-NaOH buffer (pH = 9.5), 0.2 mM NAD and 20 µg of cell homogenate protein. Reaction was initiated by the addition of 10 µL of 0.25 U β-hydroxybutyrate dehydrogenase [27].

Lactate level was determined using NADH / NAD conversion technique, at λ = 340 nm and 37 ^{o}C. 1 mL of reaction buffer contained 0.08 M glycine-NaOH buffer (pH = 9.5), 0.2 mM NAD and 20 µg of cell homogenate protein. Reaction was initiated by the addition of 10 µL of 4 U lactate dehydrogenase [27].

Malate levels were determined using NADH/NAD conversion technique, at 340 nm and 37 ^{0}C. 1 mL of reaction buffer contained 0.1 M glycine-NaOH buffer (pH = 10.0), 0.1 M glutamic acid, 0.2 mM NAD and 100 µg of cell homogenate protein. The assay was initiated by the addition of 10 µL of 8 U MDH with 0.5 U GOT [28].

***N*-acetylaspartate** (NAA) level was assayed by HPLC method described previously [4]. Briefly, supernatant (pH = 2.5) was centrifuged at Micro Spin filter (Teflon membrane, Ø 0.22 mm, CIRO, Cat# CIPT-02). 90 µL of supernatant (90 µg of cell homogenate protein) was analyzed in a Synergy 4u Fusion RP 80A column (250 x 4.6, Phenomenex, Cat# 00G-4424-EO) by HPLC A-200 system (Perkin Elmer). The assay was performed under isocratic conditions (mobile phase: 0.1% H_3PO_4 / 1% methanol / water, pH = 2.5) with flow rate: 0.9 mL/min and separation time: 20 min; t_{NAA} = 7.0 min (λ = 210 nm) [4].

Oxaloacetate and **pyruvate** levels were determined using NADH/NAD conversion technique, at 340 nm and 37 ^{0}C. 1 mL of reaction buffer contained 0.1 M Tris-HCl (pH=7.4), 3 mM sodium-EDTA, 0.2 mM NADH and 100 μg of cell homogenate protein. The assay was initiated by the addition of 10 μL of 1.5 U MDH (for oxaloacetate assay) or 4 U LDH (for pyruvate assay) [28].

Thiobarbituric acid reactive substances (TBARS) were used to track the lipid peroxidation products at λ = 535 nm in room temperature. Briefly, 0.5 mg of cell homogenate protein was deproteinized by 10% trichloroacetic acid in a final volume of 0.6 mL (10 min, 4 ^{o}C, gentle shaking). Next, each sample was enriched by 0.2 mL of 2% thiobarbituric acid and heated for 20 min at 100 ^{o}C [30].

Table 1S. List of compounds used in the study.

NAME	COMPANY	Cat#
0.4% Trypan Blue solution	Sigma Aldrich	T8154
1,4-Dioxan	Sigma Aldrich	292300119
2-Mercaptoethanol	Sigma Aldrich	M3148
2-aminoethyl diphenylborinate (2-APB)	Sigma Aldrich	D9754
Acetic acid	POCH	568760114
Acetonitrile	POCH	10265456
Acetyl-CoA [acetyl-1-14-C] 50 μCi	Perkin Elmer	NEC313050UC
ADP sodium salt	Sigma Aldrich	A6646
Albumin, bovine serum (BSA)	Sigma Aldrich	A4503
Amphotericin B	Sigma Aldrich	A9528
Aspartic acid L-[U-14-C] 50 μCi	Perkin Elmer	NEC268E050UC
ATP disodium salt	Sigma Aldrich	A2383
β-hydroxybutyrate dehydrogenase	Sigma Aldrich	H9408
BCIP	Sigma Aldrich	B6149
Brilliant blue G	Sigma Aldrich	27815
$CaCl_2$	Sigma Aldrich	C5426
CHAPS	Sigma Aldrich	C9426
Choline chloride	Sigma Aldrich	C7527
Chloroform	POCH	BA4431116
cis-Aconitic acid	Sigma Aldrich	A3412
Citrate lyase (CL)	Sigma Aldrich	C0897
Citrate synthase (CS)	Sigma Aldrich	C3260
Coenzyme A sodium salt (CoA)	Sigma Aldrich	C3144
$CuCl_2$	Sigma Aldrich	751944
D-Glucose	Sigma Aldrich	G5767
Dibutyryl-cAMP	Sigma Aldrich	D0627
Diethyl ether	Sigma Aldrich	309966
Digitonin	Sigma Aldrich	D5628
Dithiotreitol (DTT)	Sigma Aldrich	D9779
Dulbecco's Modified Eagle's Medium	Sigma Aldrich	D5671
DTNB	Sigma Aldrich	D8130
EDTA	Sigma Aldrich	E1644
Eserine salicylate salt	Sigma Aldrich	45720
Ethanol	Sigma Aldrich	493511
Fetal bovine serum (FBS)	Sigma Aldrich	F9665
Glucose-6-phosphate dehydrogenase	Sigma Aldrich	G8404
Glutamic-Oxalacetic Transaminase (GOT)	Sigma Aldrich	G2751
Glycerol	Sigma Aldrich	G2025
Glycine	Sigma Aldrich	G8898

H_3PO_4	Sigma Aldrich	79617
HCl	POCH	575283721
$HClO_4$	Fluka	77228
HEPES	Sigma Aldrich	H4034
HEPES sodium salt	Sigma Aldrich	H3784
IgG standard (from human serum)	Sigma Aldrich	I4506
Isocitrate trisodium salt	Sigma Aldrich	I1252
Isocitrate dehydrogenase	Sigma Aldrich	I2002
α-Ketoglutarate disodium salt	Sigma Aldrich	75892
KCl	POCH	739740114
K_2HPO_4	Sigma Aldrich	P5504
KH_2PO_4	Sigma Aldrich	P5655
K_3PO_4	POCH	742020112
$KHCO_3$	Sigma Aldrich	237205
α-Ketoglutarate	Sigma Aldrich	75892
Lactic dehydrogenase (LDH)	Sigma Aldrich	L-2500
Laemmli Sample Buffer	Bio-Rad	161-0737
L-Aspartate	Sigma Aldrich	A6683
L-Glutamate	Sigma Aldrich	G5889
L-Glutamine	Sigma Aldrich	G8540
Lithium potassium acetyl phosphate	Sigma Aldrich	0,1409
Lithium acetyl-CoA	Sigma Aldrich	A2181
Maleic anhydride	Fluka	63200
Malic acid	Sigma Aldrich	M1000
Malate dehydrogenase (MDH)	Sigma Aldrich	M2634
Mecamylamine hydrochloride	Sigma Aldrich	M9020
Methanol	Sigma Aldrich	621995156
$MnCl_2$	Sigma Aldrich	529680
N-acetylaspartic acid (NAA)	Sigma Aldrich	A5625
NaCl	POCH	794121116
NAD	Sigma Aldrich	N3014
NADH	Sigma Aldrich	N8129
NADPH	Sigma Aldrich	N1630
NaOH	Sigma Aldrich	S8045
Nifedipine	Sigma Aldrich	N7634
NH_4Cl	Sigma Aldrich	A9434
Nitrotetrazolium Blue chloride	Sigma Aldrich	N6876
Penicillin-Streptomycin solution	Sigma Aldrich	P4333
Phosphotransacetylase (PTA)	Sigma Aldrich	P2783
POPOP	Sigma Aldrich	P3754
PPO	Sigma Aldrich	D210404

Protease inhibitor cocktail	Sigma Aldrich	P8340
RNAlater®	Sigma Aldrich	R0901
SDS	Sigma Aldrich	L5750
Silicon oil AR20	Sigma Aldrich	10836
Silicon oil AR200	Sigma Aldrich	85419
Sodium oxalate	Sigma Aldrich	71800
Sodium phosphate	Sigma Aldrich	S0876
Sodium pyruvate	Sigma Aldrich	P2256
Spectra™ Multicolor Protein Ladder	ThermoFisher Sc	26623
Streptozotocin	Sigma Aldrich	S0130
Sucrose	Sigma Aldrich	S9378
TCA	Fluka	91228
Tetrabutylammonium hydrogensulfate	Sigmal Aldrich	155837
Tetraphenylborate sodium	Sigma Aldrich	T25402
Theophylline	Sigma Aldrich	T1633
Thiamine pyrophosphate	Sigma Aldrich	T4625
Thiobarbituric acid	Sigma Aldrich	T5500
Toluene	Sigma Aldrich	24529
trans-Retinoic acid	Sigma Aldrich	R2625
Tris Base	Sigma Aldrich	252859
Triton X- 100	Sigma Aldrich	T8787
Tween 20	Sigma Aldrich	P9416
$ZnCl_2$	POCH	264170113
